# How concerned significant others experience Community Reinforcement and Family Training (CRAFT) – a qualitative study

**DOI:** 10.1186/s12875-021-01596-5

**Published:** 2021-12-03

**Authors:** Rikke Hellum, Randi Bilberg, Gallus Bischof, Anette Søgaard Nielsen

**Affiliations:** 1 Unit of Clinical Alcohol Research, Clinical Institute, University of Southern Denmark, J.B. Winsløws vej 18, 5000 Odense C, Denmark; 2 Psychiatric Department, Odense University Hospital, J.B. Winsløws vej 18, 5000 Odense C, Denmark; 3 Odense Patient Data Explorative Network (OPEN), Odense University Hospital, Odense, Denmark; 4 Department of Psychiatry and Psychotherapy, University of Lübeck, Ratzeburger Allee 160, 23562 Lübeck, Germany

**Keywords:** Community reinforcement and family training, CRAFT, Concerned significant others, Relatives, Abuse of alcohol, Alcohol use disorder, Alcohol problems, Addiction substances

## Abstract

**Introduction:**

Heavy drinking causes serious harm, not only to the drinker but also to relationships and concerned significant others (CSOs). Community Reinforcement and Family Training (CRAFT) is an intervention developed to help the CSOs of substance users. The aim of this study was to investigate the drivers and aims underlying CSO participation in CRAFT, as well as their experience of the intervention itself and their module preferences.

**Method:**

This is a qualitative study based on data from semi-structured interviews with 11 female help-seeking CSOs of individuals with alcohol problems. The participants were recruited from an RCT study of a variety of CRAFT delivery formats (group sessions + written material, individual sessions + written material or self-delivered CRAFT with written material only). The interviews were audio-recorded, transcribed, and analyzed by Interpretative Phenomenological Analysis.

**Results:**

CSOs reported CRAFT helpful when both delivered by means of individual sessions or group sessions. The “Communication Element” in CRAFT, the module focusing on positive reinforcement and acquiring a clearer understanding of AUD, appeared to be particularly helpful elements of CRAFT. Furthermore, being met with acceptance and non-judgmental attitudes seemed to count highly for the CSOs. The written material a helpful supplement to the face-to-face interventions. The written material a helpful supplement to the face-to-face interventions.

**Conclusion:**

CSOs who participated in the CRAFT intervention felt helped by its components, irrespective of delivery format.

## Introduction

Alcohol problems have serious consequences for those who suffer from them; but also, for those close to the drinker, especially family members [1]. Families often experience marital problems, financial troubles, and a general feeling of insecurity because of life in a stressful environment; factors that, in sum, increase the risk of physical and mental illness [2] as well as a lower quality of life (Qol) [3].

Being related to a heavy drinking individual or to someone with an alcohol problem, i.e. suffering from alcohol use disorder (AUD), is associated with negative self-reported health, and the correlation increases with the degree of proximity to the drinker [4]. Spouses and partners of individuals who excessively use alcohol or other substances appear to experience more physical violence and aggression compared to parents [5]; and women more so than men [5–8].

Those close to an individual suffering from alcohol problems have been variously designated in the literature. In the present paper, we will use the term “Concerned Significant Other (CSO)” when referring to family members, partners, ex-partners, or friends of those with alcohol problems or who drink excessively. We will use the term “Identified Patient (IP)” to designate the individual suffering from alcohol problems and who is reluctant to seek or incapable of seeking treatment for their drinking problem.

Given that treatment utilization rates for AUDs are lower than for every other mental disorder [9], most CSOs are related to IPs who are not in treatment, and often the CSOs themselves find it challenging to seek help in stressful situations [10]. Treatment options for CSOs that are independent of treatment receipt by the IP differ in respect of their focus on improving the wellbeing of the CSO. While some interventions focus exclusively on the CSO (such as Al-Anon), others also include strategies for improving the IP’s motivation to seek treatment by teaching the CSO to apply principles of contingency management [11]. Most studies on treatment efficacy for CSOs independent of treatment receipt by the IP have been generated for the “Community Reinforcement and Family Training” (CRAFT) [12].

CRAFT aims at offering the CSO strategies and tools to use during daily contact with the IP in order to increase the likelihood of treatment-seeking on the part of the IP. Although the overall aims of CRAFT are to increase treatment engagement and reduce the drinking of IPs, CRAFT is, however, also aimed at increasing the Qol of the CSO regardless of whether the IP enters treatment or not [11].

In Denmark, CSOs can seek help and advice free of charge in most publicly funded alcohol treatment centers, although this is not always particularly well-advertised and known by the public. The support that the treatment centers offer CSOs may vary across the country, but in 2015, a Danish Clinical Guideline concluded, based on findings from a meta-analysis [13], that CRAFT was considered the most effective intervention in helping CSOs to motivate the IP to seek treatment, and the Health Authorities therefore recommended that CRAFT should be implemented in publicly funded treatment facilities [13]. Since CRAFT was an intervention strategy previously relatively unknown to the Danish treatment sector, a cluster-randomized trial was designed to investigate how best to implement CRAFT and disseminate knowledge about CRAFT to staff in the treatment facilities [14]. The present study is a qualitative sub-study of this trial.

### What does Community Reinforcement and Family Training (CRAFT) contain?

CRAFT is a cognitive-behavioral based intervention designed, through training CSOs in strategies on how to support a sober lifestyle, to motivate treatment-reluctant substance-abusing individuals to seek treatment. Moreover, CSOs are taught how they can improve their own Qol, regardless of whether their IP enters treatment or not [11]. The CRAFT intervention consists of eight modules or components:

1. Motivational strategies: Establish positive expectations by describing CRAFT in a way that increases the motivation of the CSO.
2. Functional analyses: Of the identified patient’s (IP) substance-using behavior. To outline the triggers and consequences of the IP’s use, and to use the tool to plan the CSOs intervention strategies.
3. Domestic violence precautions: Assessing the potential for violence on the part of the IP.
4. Communication Training. Teaching and practicing positive communication skills to improve communication with the IP.
5. Positive Reinforcement Training: Teaching the CSO how to use small rewards to reinforce clean and sober behavior.
6. Discouragement of using behavior/negative consequence: Teaching the CSO how to allow negative consequences in using and teaching a standard problem-solving strategy.
7. CSO self-reinforcement training/Quality of life: Exploring the CSOs’ dissatisfaction in life and evolving goals and a plan to increase CSOs’ own quality of life.
8. Suggesting treatment for the IP: Planning the best time for suggesting treatment and giving the CSO information about the treatment options available [11].

Treatment effects show the superiority of CRAFT in terms of IP treatment entry rates but equally in reducing the stress/strain on CSOs [15]. Given the strong focus on the IP entering treatment, it remains unclear to what extent CSOs find their own needs covered when being offered CRAFT – especially in the case of more recent versions that focus exclusively on treatment entry training [16]. Qualitative studies on how the CSOs experience the different modules of the CRAFT intervention, what kind of benefits they get from the CRAFT program, and which processes the CSOs are going through is under-investigated, indeed almost lacking. Using a qualitative approach can provide an in-depth understanding of their lived experience [17, 18]. Qualitative research thus has the potential to propose how a phenomenon (e.g. a finding from quantitative research) may be understood, and the aspect of most importance to qualitative research is the relevance of the explanations offered by the studies [19]. To date, only one qualitative study on CRAFT participation has been performed, focusing on a digital solution. Osilla et al. (2018) evaluated the feasibility of a web-based adapted version of CRAFT for 12 military CSOs, each living with a person who was an active duty service member or post-9/11-veteran [20]. In the study, the participants received four web-based sessions of 30–45 min. A semi-structured telephone interview was performed with the participants after they had completed all four sessions. The participants felt that the web intervention was helpful and allowed them to overcome barriers such as stigma, and to receive professional help, without anyone else’s knowledge [20].

CSOs participating in treatment may well reveal different needs and perspectives, as shown in a study on treatment motives. These include, for instance, a desire to improve their own quality of life, to be able to influence the addicted individual to seek treatment, or to motivate the addicted individual to cut down on his/her consumption [17]. Furthermore, in a study of online CRAFT, it has been shown that only a small number of CSOs eventually asked their IPs to enter treatment [21], and it is thus of interest to explore and better understand what the CSOs seek and find useful in the CRAFT intervention, i.e. how they perceive and implement the different treatment modules following the CRAFT intervention. The present qualitative study aimed to investigate the drivers and aims underlying CRAFT participation as well as experience with and preferences regarding CRAFT modules as viewed from the CSO’s perspective, based on a sub-sample of CSOs who took part in a Danish randomized controlled trial (RCT) [14].

## Methods

### Brief description of the RCT and of how the CRAFT intervention was delivered

The overall RCT study was a three-armed cluster randomized controlled trial to investigate the implementation of group CRAFT, individual CRAFT, and self-delivered CRAFT in 24 public outpatient treatment centers in Denmark. The participating treatment centers were randomized to deliver one of the three conditions only when a CSO sought help at the center. Both the CSOs in group CRAFT and individual CRAFT received six sessions and written material on CRAFT [22]. The CRAFT groups were organized as open groups with a fixed structure, where each session had a specific headline and content. Following one individual session primarily consisting of an assessment of the CSO’s situation and information about the intervention, new members could join the group at any point in the intervention curriculum until the completion of the full program. The participants who were offered group sessions also began with an individual session consisting of assessment and information before the CRAFT intervention was initiated. The individual intervention had the same content as the group intervention but was offered in more flexible order, depending on the preferences and needs of the individual CSO. The CSOs in self-delivered CRAFT only received the individual assessment and information session and the written material on CRAFT [22]. Furthermore, they were encouraged to come back after 3 months if they felt the need. It was explained to them that they would then be offered individual sessions. The primary outcome of the RCT study was to determine whether the IP entered treatment after 6 months follow-up, and secondary outcomes were changes in the IP’s alcohol intake (as estimated by the CSO), and changes in the CSO’s Qol, as measured by self-reported questionnaires at baseline, three-month and six-month follow-up. The inclusion criteria for the study were: the CSO = aged at least 18 years, being the CSO of a person with alcohol problems not in receipt of treatment, having regular contact with the IP, being willing to maintain contact with the IP for at least 90 days, and being prepared to support the IP in treatment- seeking [14]. A total of 255 CSOs were enrolled in the CRAFT study from January 2018 to December 2019.

### Design of the present qualitative sub-study

In the present study, we used a qualitative design with semi-structured interviews. We used the Interpretative Phenomenological Analysis (IPA) [18] to analyze and interpret the interviews. IPA is a phenomenological approach used to explore how people make sense of their experiences in life—in this study, understanding how CSOs experience the CRAFT intervention, what they gained from it, and how it affected their life after the intervention. IPA also involves a hermeneutic element used in the interpretation of the experiences. The researcher needs to interpret the accounts given by the participant CSOs to understand their experiences. Lastly, IPA is ideographic, which means that every case is examined in detail in order to gain insights into the precise nature of the participant’s experience. IPA tends to be applied to a limited number of participants only, because it is essential to probe each participant experience [18].

### Participants

Participants for the present study were recruited through the randomized controlled CRAFT study [14]. As a part of the six-month follow-up interview, the participants were asked if they were willing to be contacted again for further questions and information within the next 5 years. Approximately one and a half years after initiation of the RCT, we invited all participants (*n* = 40), who, at that time, had completed the six-month follow-up and had given informed consent for participation in the sub-study. We invited them by mail, telephone, or personal digital mail according to their specified preference. Fifteen CSOs accepted the invitation to participate in the present qualitative study. Three of the participants, however, did not accept the invitation until after completion of the first 12 interviews, and at that time, it was considered that data saturation was reached, since no new information or themes emerged in the last interviews. One audio file turned out to be damaged and could not be transcribed ad verbatim. Hence, the present study is based on interviews of 11 female CSOs: four participants in group CRAFT, five in individual CRAFT, and two in self-delivered CRAFT. We gave the participants oral and written information about the study and their rights, after which they signed a statement of consent.

The sample of participants in the present study consisted of 11 female CSOs of persons with alcohol problems. Six CSOs were living with their drinking boyfriend/husband, two CSOs had a drinking ex-husband (neither cohabiting anymore), one had a drinking boyfriend (not cohabiting), one had a drinking brother (not resident at the same address), and one had a drinking stepson (not resident at the same address). All IPs were men. The CSOs were between 29 and 60 years old (mean age: 51). The CSOs had known the IP for 2–43 years (mean: 23.5) and had had the supporting role concerning the IP for approximately 2 to 30 years (mean: 12 years). Participant’s characteristics are displayed in more detail in Table 1.

**Table 1:** Characteristics of the sample

ID:	Age (years)	Type of CSO	Cohabitant / Not cohabitant	Know the CSO in (years)	The supporting role concerning the IP in (years)	Type of CRAFT intervention
1	57	Wife	Cohabitant	38	15	Group
2	54	Wife	Cohabitant	28	28	Individual
3	29	Girlfriend	Cohabitant	2	2	Group
4	52	Ex-Girlfriend	Not cohabitant	12	3	Individual
5	57	Wife	Cohabitant	25	25	Individual
6	59	Stepson	Not cohabitant	37	13	Group
7	53	Ex-Girlfriend	Not cohabitant	13	5	Written material
8	46	Girlfriend	Not cohabitant	5,5	5,5	Written material
9	43	Sister	Not cohabitant	44	5	Individual
10	48	Wife	Cohabitant	27	3	Individual
11	60	Wife	Cohabitant	24	5	Group

Overall, the 11 CSOs who accepted the invitation to participate were fairly representative of the RCT participants as a whole in terms of age, gender, type of relationship to the IP and type of CRAFT intervention received.

### Interviews

The semi-structured interview guide (see Table 2) consisted of a few demographic questions and a series of open questions within two areas: the stresses and strains due to the IP’s problematic drinking (findings reported in another sub-study), and their experience with CRAFT. We performed the 11 face-to-face individual interviews in autumn 2019. The interviews took place in a private and secluded place, for example in the participants’ homes, workplaces or in the treatment facility where the participant had received the CRAFT intervention. The interviews were conducted by the first author and supervised by the experienced interviewer and last author. All interviews began by encouraging the participants to be truly honest and open about the intervention they had received, thus providing highly pertinent feedback to the interviewer, who had taken part in planning and evaluating the CRAFT intervention. Thus, if the participants had any reflections on the experience that they felt might possibly improve the intervention they were encouraged to share them with the researcher. Afterwards, the participants were asked about basic demographic information before being encouraged to talk about the experience of the CRAFT intervention. All interviews were recorded on a digital voice recorder and lasted between 20 and 62 min with an average of 41 min.

**Table 2:** Semi-structured interview guide

Opening question	Can you try to explain what you experienced from the time you realized there was an alcohol problem until you sought help?
	How did you experience receiving the CRAFT counselling?
	Did you find that it made a difference? What was it that made a difference?
	When did you notice a difference? Was it immediately or after some time?
	What was the best thing you got from the counselling?
	Were there some elements of the counselling that you did not find useful?
	What more could you have wished for from the treatment?
	How did you find the written material (the book)?
To those who received individual CRAFT	How did you find the individual counselling?
To those who received Group CRAFT	How did you find being in a group?
To those who received the written material only	How did you get on with the written material? Was it adequate?
	Do you feel that participating in CRAFT has changed the atmosphere/interaction between you and the person you support? What has changed?
	Do you think the person you support has changed at all? If so, has he/she commented on this? What happened?
	Have you received any other kind of help relating to the person’s alcohol use? – If so, what kind of help? Have you received help from family, friends, job, your/his social network? What happened?
	Is there anything else important that you’d like to mention that I’ve missed?

### Data management and analysis

The audio files were fully transcribed by the first author and two student assistants in the software program Nvivo 12 PRO. The first author anonymized and quality-controlled all the interviews. Ten participants wished to read the interviews, so the transcripts were sent to them for validation and comments. None of the participants had comments on the transcripts. An IPA inductive approach was used for the analysis [18].

Completed by two researchers, several-step analyses were applied to ensure high quality. First, the interviews were read several times while taking notes and adding descriptive comments. Afterwards we developed themes by discussing the findings within each interview until no new themes emerged and thematic saturation accrued [23], whereupon we searched for connections across the emergent themes. This was done for all interviews and alongside the interview process. Finally, we looked for patterns across all the interviews [18]. The quotations in the results section are followed by an ID number, the role of the CSO (e.g. wife, girlfriend or stepmom), whether resident at the same address as their IP or not, and the intervention received (individual, group or self-delivered). The interviewer is identified as “I”.

## Results

The analysis resulted in six overall categories and two sub-categories.

1. Entering the CRAFT program
2. The CRAFT Components
3. The format in which the CRAFT intervention was delivered
  The written material on CRAFT
4. What was gained from the CRAFT intervention? How well did it work?
5. What were the participants’ perceptions of the CRAFT intervention?
6. Filling out questionnaires
7. Suggested improvements to the CRAFT intervention

At the end of the result section we present a model of the “Helpfulness of the CRAFT components”.

### Entering the CRAFT-program

Most CSOs reported that, on hearing about the CRAFT program, they were very enthusiastic. One of the CSOs described how she heard about the CRAFT study from a friend:

*So, I contacted the treatment center right away and was completely hooked on it – because I was out of my depth! I couldn't find any tools at all that could help me. And if I could do something, both for you who are involved with this project, and at the same time for myself, well it would be a complete win-win, after all. So, I had no doubt that it was something for me.*

*(10, wife, cohabitant, individual)*

Entering the treatment center was transgressive for some of the CSOs and a positive experience. They felt very well received both in the treatment centers and when they began the CRAFT intervention. The CSOs described how they felt comfortable around and accepted by the treatment providers, which was very important for them. They felt they were met without any prejudice or sense of taboo. For several of the CSOs, it was the first time they had ever spoken to anyone else about their IP’s alcohol problems; hence just describing their situation gave them a feeling of relief. Moreover, they explained how pleasant it was to be met by a person who took their worries seriously, validated their hunches about the alcohol problems, and encouraged them to hold on to those feelings.

CSOs in all three intervention groups also mentioned how they came to realize that they were not alone in their situation. This aspect in particular meant a lot, as they had often felt very lonely, as one of the CSOs explained:

*8: “But, but they have, perhaps, been good at sharing - the fact that I am not the only one with this issue”*

*I: Could you try to describe what this means; what it’s been like having this feeling of being alone with it?*

8: It’s, it's hard to hang in there all the time, though, and, and, and... You just want affirmation that it's okay. It's okay to, uh, that you, uh, sometimes want to pack it all in. And to say F*ck you. This feeling is okay to have too. The feeling of constantly wanting to feel normal.

*(8, girlfriend, not cohabitant, self-delivered)*

Only a few of the CSOs had informed their IP beforehand that they were going to seek help. One CSO explained how participating in CRAFT was “her thing”:

*“Well, I didn’t tell my husband that I have been taking part in this. I haven’t needed to, really”.*

*(10, wife, cohabitant, individual)*

### The CRAFT components

As outlined in the introduction, the CRAFT program comprises eight components. In the interviews, the CSOs referred to these components as tools and reflected that it was great to be getting such useful instruments, though they felt that some were more relevant than others. The most valued CRAFT component was “Communication Training.” Almost all the CSOs commented on how helpful the communication training had been. By learning to communicate more clearly and precisely, training through role-play with the therapist before communicating with the IP, and learning how to communicate with the IP, they experienced remarkable improvements in their communication with the IP. One of the CSOs explained how she even felt communication with the IP change:

*“But then to learn how to speak up, even about the fact that I, just … when I had to communicate things to my husband, he just saw me sitting there beating around the bush, and explaining blablabla, and in the end we just got our wires crossed because, well, it was unbearable to listen to. So, so there I kind of discovered that it does pay off in these contexts to be very precise in articulating what I want”.*

*(5, wife, cohabitant, individual).*

Moreover, the CSOs reported that they had become better at staying calm and keeping to their “own half of the course” when communicating. The effort they had put into communication training led to less confrontation, and one CSO noticed how her husband began to take note of the things she said when she spoke more incisively. Other CSOs realized that it had become easier to talk about difficult subjects and problems. Some CSOs mentioned how they found that their IP became more open when the CSOs themselves switched to a more positive communicative style. It also became clear to the CSOs that it was challenging to communicate when their IPs were drunk and that it was impossible to persuade them to stop drinking once a drinking session had begun.

Another CRAFT element that also made a particularly big impression on the CSOs was “Positive Reinforcement Training.” The rationale behind the strategy of making a sober life more attractive than a life dominated by drink had been considered by some of the CSOs even before entering the CRAFT intervention, but it was a kind of revelation for others. One CSO explained how she realized that she had previously detached herself from her stepson, but after the CRAFT intervention, she made a point of giving him more positive attention when he was sober:

*“So, I have practiced being present for him and paying attention to what interests him. And he is talented in many ways, and many things work for him. And by creating a distance between us, I achieve nothing at all”.*

*(6, stepmom, not residing at the same address, group)*

Other CSOs described how they realized that for a long time they had almost entirely focused on the negative aspects of their life together with the IP. Most found that by focusing on the positive things and praising them, they could increasingly come to value and embrace the more positive sides of their IP. A few, however, found it difficult to affirm the positive sides of their spouse/partner when sober, given, as one CSO put it, that he had caused his family so much harm and sorrow. Another CSO described how her boyfriend was taken aback when she began to praise the person he is when sober.

The CRAFT component “Focusing on Own Quality of Life” was also highlighted and found helpful by the CSOs. The focus on the CSO’s own quality of life came as a surprise to some. Still, most explained how they had become better at prioritizing themselves and being good to themselves as a result of the intervention. One CSO explained how she realized that it was important to her to be loaded up with positive things:

*“ … This…having the energy to think about what can you do that’s good for yourself. So, you can refill your energy tank, and also be better empowered to stand up to him, if I can put it like that. I've probably not been very good at doing that”.*

*(10, wife, cohabitant, individual)*

The CRAFT component “Negative Consequences” was also explored during the interview with the CSOs. Most had found that allowing negative consequences of the drinking to happen made a huge impression on their IP, for instance, no longer being able to see the children or grandchildren when drinking. Several of the CSOs had begun to simply withdraw from their IP when he was drinking. One CSO experienced that her quality of life increases when she withdraws from the IP and another CSO explained how she began to communicate more plainly to the IP, letting him know how unpleasant he was to be with when drinking:

*10: Yes and of course, change my behavior and make it clear to him. When he was nice to be with and when he was not nice to be around. And choose to stay around him or not*

*I: Can you try to explain how you did that?*

*10: Well, it was to put it into words, not just leave when he came home or came in and was under the influence, but also to explain why I left*

*(10, wife, cohabitant, individual)*

However, not all CSOs found it easy to make use of negative consequences in daily life; in particular when the consequences affected their common home. As one of the CSOs noted, she had to be able to live in the home as well.

The CRAFT component “Functional Analysis” was overall found to be a very helpful tool, contributing to a better sense and understanding of the drinking context, and, thus, helping to give the CSOs a kind of map indicating when it was possible to intervene. One CSO, though, did not find it helpful at all.

### The format in which the CRAFT intervention was delivered

How the CRAFT intervention was delivered was also addressed in the interviews. Overall, the five CSOs who received “Individual CRAFT sessions” were satisfied with the intervention. They appreciated the flexibility of the program and felt that it met their needs. One CSO, however, did not feel that six sessions were sufficient to work through all her problems and feelings, and some CSOs would have liked to discuss issues with other CSOs in similar situations.

The four CSOs among the participants in the present study who received CRAFT in group sessions, were overall satisfied, but one CSO would have preferred individual sessions of CRAFT because she felt she needed more focus on her own situation. Still, they also mentioned that they would have wished for more group members (one group consisted of only a few members). The CSOs who had received CRAFT in group sessions stressed how much they appreciated conversing with and seeing other CSOs, with same kind of problem as themselves, since this left them feeling less alone with their own challenges. It also contributed to a more varied picture of alcohol problems. The participants found the open-group format a distinct plus since it allowed the CSO to enter the group without any waiting time and also because the group thus became even more diverse. Several of the CSOs experienced that other CSOs were way worse off than themselves and that put their own experiences in perspective, as one CSO explained:

*“I felt better about myself the days when she - the other one – was with me. Because I could hear that she was much worse off than me. I could go home and think, wow, okay, perhaps it’s not so bad here at home.*

*(3, girlfriend, cohabitant, group)*

Some CSOs found it easier to help solve the other group members’ problems in the sessions than propose solutions to their own. When entering the group, some of the CSOs were anxious to meet somebody familiar in the group. However, the CSOs described how they nevertheless had taken firm decisions beforehand about being honest about their situation, for once. One CSO explains:

*“It was my experience that what is said here stays in this room. And if I am not honest in this room…. And I can totally trust that the people who hear this, of course, they are also bound by confidentiality - which was mutual for the woman who sat opposite me”.*

*(6, stepmom, not cohabitant, individual,)*

Two of the CSOs participating in the present study received the written material as the only source of information and help. Both indicated that the written material was helpful and supportive:

*“I have that book next to my bed and, um, I often read it. Probably, in fact, to get the advice from it. And just like being affirmed in that what I do is actually good enough, or it is OK enough. In that way, I think it is really great”.*

*(8, girlfriend, not cohabitant, self-delivered)*

The CSOs felt a positive change very soon after reading the book:

*“Well, I think it happened pretty quickly. Because I read it immediately after I got it. Eh. It didn’t take me long. And that’s where I started, sort of like – to be able to see things and do things”.*

*(7, ex-girlfriend, not cohabitant, delivered)*

*“Well again, to get to know myself and know and find out what I am willing to be a part of and what I cannot. And also, how I can best help? Um. I think that has actually been good. Because a lot of changes have happened since”.*

*(8, girlfriend, not cohabitant, delivered)*

However, the CSOs who only received the self-delivered CRAFT did feel somewhat on their own and missed more personal contact with the treatment provider or other CSOs. They were inclined to think that they would have improved faster if they had had a more personal connection with others. Despite this, no CSOs returned to the treatment centers after 3 months to ask for additional individual support.

### The written material on CRAFT

Not only the CSOs who relied solely on the introduction session and the written material, but also the CSOs who received the same written material in addition to the individual sessions or group sessions, were happy with it. There was a shared satisfaction with it and all found it very readable, informative and understandable. The CSOs explained how they felt seen and affirmed when reading it. They felt validated in their assumptions of the IP’s drinking too much, and they felt affirmed in their sense of doing the right things when they started using CRAFT. Several CSOs described how they felt recognized in the material, and one CSO even said that she could have been the one writing it. The CSOs found it very useful to have read the written material before participating in the sessions, as a form of preparation. Others also used the material to brush up on the components after sessions.

### What was gained from the CRAFT intervention? How well did it work?

The CSOs had very different experiences of how quickly they made use of the strategies and components learned from CRAFT, and what they felt worked. One CSO felt immediate relief from the moment she stepped into the treatment center and openly described her situation to the treatment provider. Another CSO also registered immediate changes because she found the components of CRAFT highly useable, in addition to the support received from the treatment provider. One of the CSOs who had received self-delivered CRAFT, also described an immediate change after reading the written material because it helped her improve communication with her IP. It took her somewhat longer to master the rest of the CRAFT components. Other CSOs described how it took a few months before they experienced a positive change in their lives. One even described how, after almost a year, she still kept improving. Learning the strategy of focusing on the positives rather than the negatives was described as refreshingly new and particularly helpful. A few CSOs had felt tempted to give up during the early stages of the intervention due to an overwhelming feeling of hopelessness but began to improve after a few months. Thus, the path of improvement varied among the participants.

A particular outcome of participating in the CRAFT intervention, stressed by the CSOs, was becoming better at withdrawing from the IP when necessary. When the CSOs gained a better understanding of why the IP behaved as he did, and of how the alcohol use disorder works, it also became easier for them both to see things from the IP’s perspective, and how it differed from their own. For many of the CSOs it was really an eye-opener or indeed a wake-up call to discover what alcohol did to the drinking IP. One of the CSOs explained:

*“Yes, that understanding of him, also the fact that, well I think it was a revelation. I can remember the example with the scale model and how to find out, well, what it really is that motivates him to drink, what it is that motivates him not to drink, that’s the way it is, and where I just actually hurt inside, because it is like it is, that there simply wasn’t very much that motivated him not to drink during that time. So, I can easily see the mechanism, hmm, that is part of him, and that understanding, and seeing it from his perspective, hmm, not a bad thing to have, I think”.*

*(4, ex-girlfriend, not cohabitant, individual)*

Gaining this understanding of alcohol use disorder helped the CSOs realize that the IP did not drink because of them, and it was not their fault that he started drinking (again). This differentiation between themselves, the IP, and the alcohol use disorder also made the CSOs become more independent as individuals. It became easier for them to stand a bit back from the drinking behavior and make decisions for themselves. Several CSOs described how it became easier to draw a line or make demands regarding what could be expected in the relationship. One CSO explained how she began to take control:

*“Well, I feel that it is like, it has, at least, been an eye-opener that was about uh, you cannot control that, my friend. You cannot control me. I am the one who decides what I do”.*

*(5, wife, cohabitant, individual)*

Some of the CSOs found that their efforts with CRAFT led to changes in the IP’s drinking pattern. Hence, they observed that their time together became better and desire for him increased when he did not drink.

### What were the participants perceptions of the CRAFT intervention?

The CSOs perceived their own outcomes from participating in CRAFT differently, depending on their role as a CSO (wife, girlfriend, stepmom), and the format for the CRAFT intervention. Some of the CSOs were able to use the CRAFT strategies to motivate their IP towards treatment, but most of the CSOs reflected that participating in CRAFT had improved their quality of life and the relationship with the IP independently of whether he entered treatment or not. Four of the CSOs participating in the present study indicated that their own outcome of CRAFT had been positive despite realizing during the intervention that their loved one probably would never quit drinking.

Some of the CSOs decided to leave their drinking husband or boyfriend, either immediately before or after the CRAFT intervention. Even though their relationship with the IP came to an end, they felt they had achieved a higher satisfaction with life, and that the relationship with their ex-husband or boyfriend was improved due to the CRAFT intervention. As one explains:

*“So, I think I already had – I have some good tools for how, even when we were apart, how… well I think it actually ended up fine and we have always been able to work things out, also regarding our son, it has been very constructive”.*

*(4, ex-girlfriend, not cohabitant, individual)*

Overall, almost all participants described the CRAFT intervention as helpful:

*CRAFT has been a gift (2)*

*CRAFT is goal-oriented (5)*

*CRAFT brought me to where I am today (7)*

*So, I got an energy boost. So, it has helped (9)*

### Filling out questionnaires

The participants in the present study were recruited from the group of participants in the larger RCT, and so had filled out questionnaires at baseline when entering the RCT, after 3 months (end of intervention), and again at the six-month follow-up. During the interviews for the present study, several of the participants brought up the questionnaires as a theme in spite of the fact that this was not a part of the interview guide. The participants reflected how filling out the questionnaires had acted as a reminder and helped them focus during the process. Moreover, they felt that the questionnaires helped them in summing up the intervention and they felt affirmed in doing the right things, as one explained:

*“But I also think that, subsequently, the questionnaires I was given, they helped me hold on to the notion that it was not a just a course I was attending, but that it is actually something continuing. I have actually thought that. And that is also why I say yes to this (interview red.), because it wasn’t just a course and that is just how it is. After all, it's something for life, for sure. It (alcohol red.) is a part of our family”.*

*(6, stepmom, not cohabitant, group)*

Most notably the two CSOs from self-delivered CRAFT talked about the questionnaires as if they had been part of the intervention:

*“Well, the questions did mean that you were, like, affirmed in some things. And, and it was not wrong to write, well, no one else would know what I was doing, at all. Nobody would know about it, right? So, I could just write what I wanted to write”.*

*(7, ex-girlfriend, not cohabitant, self-delivered)*

### Suggested improvements of the CRAFT intervention

Several of the CSOs suggested adding a follow-up session with a treatment provider 4 months after the intervention, either face-to-face or delivered via a telephone/video call. It was further suggested that such a follow-up or after-care session might prevent a feeling of abandonment, would be helpful in the face of new challenges that have emerged and which it would be salutary to discuss. But also helpful in terms of providing feedback on the continuing process – whether or not the CSO was managing well. The CSOs who had young children also noted that they would have appreciated more focus on how best to support the children in their families. One CSO felt that the program was too focused on getting the IP into treatment which she had already realized was not going to be possible.

### Helpfulness of the CRAFT components

Based on the analysis of the interviews referenced above, we created a tentative model of how the different CRAFT strategies and components might function in terms of potential treatment-seeking on the part of the IP and increasing the quality of life for the CSO. Elements such as “Domestic Violence Precaution” were not mentioned during the interviews despite the fact that this element had always been part of the CRAFT intervention and is highlighted in the written material. The CSOs participating in the present study were not facing violent behavior, and this strategy is thus not included in the figure. Nor were “Motivational Strategies” directly addressed during the interviews as a theme, probably since the motivational methods are already embodied by the therapist’s style and the initial session introducing the CSO to the intervention. They are essential to clarifying the goals in relation to the needs of the CSOs and what the CSO can expect from the intervention.

**Fig. 1:**
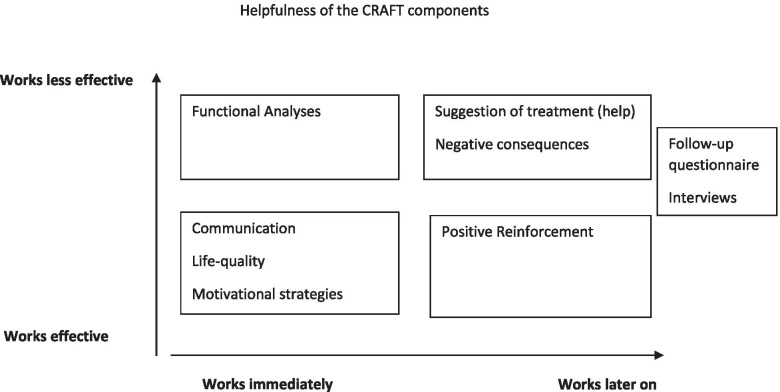
Model on how the different CRAFT strategies and components may function in relation to the treatment-seeking of the IP and the increase of the quality of life for the CSO

The “Functional Analysis” was only referred to by a few participants and described as creating an overview useful in applying other CRAFT strategies. “Life-quality,” “Positive Reinforcement,” and “Negative Consequences” were appropriate strategies for the CSOs, and their experience was that some of them were easy to implement and worked immediately, where others were more complicated to use and harder to learn, such as “Positive Reinforcement” (Fig. 1).

## Discussion

Overall, we found that the CSOs considered the communication component of CRAFT to be a particularly helpful part of the intervention. Thus, our findings confirm those of the previous qualitative study of CRAFT participation [20]. It was in switching communication styles and focusing on the positive aspects with the IP that the CSOs experienced an enhancement of their relationship with their IP and with life in general. CSOs also found it helpful to gain a better understanding of their IP’s disorder. This finding matches the review of Gammage et al. (2020), who found that people living with persons with mental health problems reported better Qol, better communication, and better family relationships once they had acquired a deeper understanding of the disorder in question through, for instance, psychoeducation. A better insight into the relevant disorder was associated with improvements in understanding, coping, and stigma reduction for the relatives [24], and this corresponds with the findings of the present study. Improving the quality of infra-family relationships is essential since it seems to impact the substance problems and is related to positive treatment outcomes [1, 25].

The CSOs shared how several components learned from participating in CRAFT induced an increase in their quality of life. Especially when the CSO learned to separate themselves to a greater extent from the IP and when they learned that it was acceptable to prioritize their own interests, they registered an improvement in their quality of life. This may partly be because the CSOs to a greater extent began to focus more on own needs and to a higher degree doing things for themselves that gave them pleasure and joy and directly improved their Qol; but it may also be because the increased focus on own needs rather than the needs of the IP that led to change in the relationship with the IP that impacted on the behavior of the IP positively. Whether the mechanism is the one or the other, the phenomenon has been described before. Gammage et al. (2020), for instance, found that when CSOs focused more on their own personal relationships than on the caregiving relationship, they reported higher personhood [24]. Copello et al. (2005) explain that *“A family member cannot stop individuals from drinking, but they can change their own behavior in a way that will help the IP recognize that behavior is problematic and make it favorable to change behavior”* [1]. This might explain why changes may happen even when the focus in the interventions aimed at the CSOs does not have treatment entry of the IP as an immediate goal. The changes may occur when the CSO changes behavior and focuses on their own interests rather than exclusively on the IP.

CRAFT is known to be a very structured intervention focusing on applicable tools; hence the time allowed for the CSO to talk freely and in detail may be limited to some extent, a critique of CRAFT that previously has been raised. Orford et al. (2013) criticized interventions like CRAFT for lacking a clear focus on the CSO’s needs [26]. However, the CSOs in our study indicated that they felt their needs were met in all three types of intervention. Moreover, they felt that their own Qol was highly prioritized in both the CRAFT intervention and in the written material they received, which opened windows for them. This may be because the treatment providers had a clear sense of CRAFT involving not only giving tools to the CSO to increase their likelihood of motivating the IP to seek treatment but also, as an equally important aspect, of increasing the Qol of the CSO. In both the intervention manuals and the written material, it was explained in detail how being a significant other to an individual with alcohol problems takes its toll. Emphasis was laid on the necessity of focusing substantially on own wellbeing in order to improve it as much as possible, and it was pointed up that increasing own wellbeing was fundamentally key to creating change in the overall situation, i.e. the drinking and treatment-seeking by the I.P.

The group CRAFT and the individual CRAFT conditions in our study consisted of 6 sessions, which is of a shorter duration than that of the interventions that have previously been offered in research studies. Overall, the participants in the present study were satisfied, but some of the CSOs felt that the time allocated to the intervention was too limited. Several of the CSOs suggested a follow-up session 1–3 months after the conclusion of the intervention. The questionnaires used in the RCT study at baseline, three-months, and six-month follow-up were mentioned several times by the CSOs. The two CSOs in the self-delivered CRAFT condition, in particular, seemed to directly benefit from filling out the self-reported questionnaires. This raises the question of whether the determining factor is the reception of the actual intervention or participation in the study itself. A review by Kramer Schmidt et al. (2018) found that research assessments and, thus, research projects in general, may influence the outcome in studies of psychosocial treatment for alcohol use disorder [27]. This may also be the case in studies of interventions aimed at CSOs. It may be the recurrent assessment by means of filling out structured research questionnaires in the present study functioned as a form of self-monitoring of own wellbeing and of the relationship to the drinker over time that allowed for recognizing progress. The research follow-up questionnaires may thus have functioned as a helpful, integrated part of the intervention.

Independent of how the CRAFT intervention was delivered, all the CSOs reported feeling helped, in some way or another, by the CRAFT program, but probably particularly if the intervention consisted of sessions with a treatment provider. The two CSOs who received self-delivered CRAFT felt adequately helped, but they also felt a bit out on a limb and missed having someone with whom to discuss their problems. This indicated that written material, while being of great help, was probably not as helpful on its own as when offered in combination with sessions with a professional.

Some CSOs in the present study were capable of using all the CRAFT components, while others found it difficult, particularly in the beginning. The ability to use all the CRAFT components might be linked to how chaotic the CSOs perceived their own situation as being. Those who were capable of using the CRAFT components seemed to move on in their own trajectory, either by staying with the IP or ending the relationship. The CSOs seemed to experience an increase in life quality regardless of whether the IP entered treatment, and regardless of whether they stayed with the drinking person. This underlines the importance of a thorough clarification of goals when beginning the CRAFT program. This finding is in line with the effects of the CSO’s treatment motivation on CRAFT outcomes, as outlined in another study [17].

With this study we contributed more in-depth knowledge about how a CRAFT intervention, delivered in three different formats, was perceived and experienced by CSOs of persons with alcohol problems resident in Denmark. A central issue for qualitative research is to be able to understand and explain the phenomena that occur. The interviews of the CSOs in the present study gave us an understanding not only of how participating in CRAFT was perceived and which elements were deemed pivotal, but also that participation was found to increase own wellbeing and Qol, an increase that was ascribed to enhanced knowledge, access to tools and, in particular, to a feeling of not being deviant or alone. These new insights afforded by the CSOs’ perspectives on the intervention will feed into the planning of studies and implementation of unilateral interventions like CRAFT in the future.

Important findings from this study include the fact that being met with acceptance and in a non-judgmental manner appear to be helpful, regardless of whether the CRAFT components were delivered in individual sessions or group sessions. Even self-delivered CRAFT, in the form of written material, was considered helpful, due to its ability to mirror the situation of the CSOs in a way that allowed them to realize that they were not alone or deviant. In fact, the written material may for some function as a helpful intervention in itself, albeit probably not as effective and helpful as in combination with individual or group sessions. The “Communication Element” in CRAFT, positive reinforcement, and knowledge about the disorder were, in particular, highly rated by the participants in the present study. In light of the suggestions from the CSOs related to how they viewed the impact of participating in follow-up interviews (which were part of the research and not the intervention per se), it might be relevant to extend the CRAFT intervention to include a further session, phone call or perhaps even a written contact a few months after its conclusion, as a further brush-up or follow-up session.

### Strengths and limitations of the study

This study is the first qualitative study of CSOs participating in a non-web-based CRAFT intervention. Thus, this study contributes important findings that can be used in the planning of future interventions aimed at CSOs. Some limitations should, however, be noted. Of the 40 CSOs from the RCT study that we invited for a further interview, only 15 were willing to participate. Of the 11 interviews we included in the study, we ended up having interviews with only two CSOs from the control group, i.e. CSOs having received written material only. Although data saturation was considered reached and the participants were a relatively representative sub-group of the RCT participants, we cannot generalize these 11 CSOs’ experiences to all the CSOs. It is likely that the CSOs who were less satisfied with the treatment did not sign up for this study. After the interview, several of the CSOs who participated in this study indicated that they were pleased to have done so, even viewing the interview itself as a kind of additional session. We cannot rule out that these CSOs had a special interest in participating in the interview, because the alcohol problems were still very much present in their lives. Moreover, only female CSOs accepted the additional interview. The two participants in the self-delivered CRAFT condition were overall satisfied with the intervention. This might not, however, be the whole picture since only two participants from this group were willing to be interviewed. Lastly, the interviewer is part of the research group that planned and evaluated the overall RCT study, and has, therefore, followed all the phases of the project. However, during the interview the CSOs were consistently encouraged to be honest and open and to express all their thoughts about the CRAFT intervention they had received, whether positive or negative. It was explained to them that the thoughts and reflections of the CSOs would be of great value to the future planning of the development of meaningful interventions.

## Conclusion

This study showed that the CSOs of people with alcohol problems who participated in the CRAFT intervention and in the present study, feel helped regardless of whether the CRAFT components are delivered by means of individual sessions or group sessions. Self-delivered CRAFT, in the form of written material, was considered helpful alongside the interventions and may even, for some, function as a helpful intervention, albeit probably not as effective. The “Communication Element” of CRAFT, Positive Reinforcement, and a better understanding of the nature of the disorder appeared to be particularly helpful.

## Data Availability

The datasets used and/or analyzed during the current study are available from the corresponding author on reasonable request.

## Abbreviations

CRAFT: Community Reinforcement and Family Training
CSO: Concerned significant other
IP: Identified Patient
Qol: Quality of life
RCT: Randomized control trial
